# Effects of Culture Period and Plant Growth Regulators on In Vitro Biomass Production and Phenolic Compounds in Seven Species of *Hypericum*

**DOI:** 10.3390/plants14152437

**Published:** 2025-08-06

**Authors:** Doina Clapa, Monica Hârţa, Ana Maria Radomir, Adrian George Peticilă, Loredana Leopold, Floricuţa Ranga, Dorin Ioan Sumedrea

**Affiliations:** 1 Faculty of Horticulture and Business in Rural Development, University of Agricultural Sciences and Veterinary Medicine of Cluj-Napoca, 400372 Cluj-Napoca, Romania; doina.clapa@usamvcluj.ro; 2 National Research and Development Institute for Biotechnology in Horticulture Ștefănești-Argeș, 117715 Ștefănești, Romania; radomir.anamaria@yahoo.com (A.M.R.); dsumedrea@yahoo.com (D.I.S.); 3 Faculty of Horticulture, University of Agronomic Sciences and Veterinary Medicine of Bucharest, 011464 Bucharest, Romania; apeticila@gmail.com; 4 Faculty of Food Science and Technology, University of Agricultural Sciences and Veterinary Medicine of Cluj-Napoca, 400372 Cluj-Napoca, Romania; floricuta.ranga@usamvcluj.ro

**Keywords:** benzyladenine, bioactive compounds, hypericin, HPLC, naphthodianthrones, shoots culture

## Abstract

This study evaluated biomass accumulation and phenolic compound production in seven *Hypericum* species (*H. androsaemum*, *H. calycinum*, *H. hirsutum*, *H. kalmianum*, *H. olympicum*, *H. perforatum*, and *H. triquetrifolium*) cultivated in vitro under varying growth regulator treatments and culture periods. Shoots were grown on Murashige and Skoog (MS) medium supplemented with benzyladenine (BA) or meta-topoline (mT) and analyzed after 40 and 60 days. MS medium supplemented with 0.2 mg/L BA was the most effective condition for promoting biomass across all species, with shoot fresh weight increasing significantly at 60 days, particularly in *H. olympicum*, *H. perforatum*, and *H. triquetrifolium*. High-performance liquid chromatography coupled with diode array detection and electrospray ionization mass spectrometry (HPLC-DAD-ESI-MS) identified 13 phenolic compounds, including flavonols, hydroxycinnamic acids, anthocyanins, phloroglucinols, and naphthodianthrones. Phenolic profiles were species-specific and influenced by culture period. *H. kalmianum* accumulated the highest total phenolic content (37.6 mg/g DW), while *H. olympicum* was the top producer of hypericin and pseudohypericin. These results highlight the crucial role of culture conditions in regulating both biomass and phytochemical production and provide a promising approach for producing bioactive metabolites in *Hypericum* species through in vitro systems.

## 1. Introduction

Medicinal plants have always played a fundamental role in traditional and modern medicine and are extensively utilized in pharmaceuticals, cosmetics, and nutraceuticals [1]. The growing demand for plant-derived products has driven the need for sustainable strategies to increase production while preserving natural habitats. In vitro cultures serve as alternative sources for producing biomass free of biotic and abiotic contaminants [2,3,4].

The *Hypericum* L. genus (*Hypericaceae* family) has a nearly global distribution and is widely recognized for its significant medicinal potential, attributed to the presence of unique groups of secondary metabolites [5,6,7]. Although *Hypericum* species are known for their significant pharmaceutical value, in vitro techniques for biomass production and the isolation of bioactive compounds have only been applied to a limited number of recognized species.

Over the past decade (2015–2025), a wide range of in vitro strategies have been employed across several Hypericum species, particularly *H. perforatum*, to enhance biomass production and the accumulation of secondary metabolites. These include shoot, callus, root, and cell suspension cultures, often coupled with elicitor treatments, each aiming to enhance biomass production and/or metabolite yield.

Among these, shoot cultures remain the most widely studied system. For example, cultivars ‘Elixir’, ‘Helos’, and ‘Topas’ were micropropagated on MS and LS media supplemented with 0.1 to 2.0 mg/L BA and NAA, resulting in robust shoot multiplication [8,9,10]. Shoot cultures of *H. perforatum* on MS medium supplemented with 0.05 mg/L NAA and 0.5 mg/L BA have also been used for the accumulation of bioactive compounds [11]. Similarly, *H. richeri*, *H. tetrapterum*, *H. calycinum*, *H. aviculariifolium*, *H. pruinatum*, and *H. amblysepalum* responded positively to BA, IBA, and GA_3_ combinations [12,13,14].

Callus induction was frequently achieved using 2,4-D in combination with BA, TDZ, KIN, or picloram, either on MS, B5, or LS media. For instance, Ebadollahi et al. [15] applied 1.0 mg/L 2,4-D and BA, enhanced with perlite or TiO_2_-perlite nanocomposites, while Eray et al. [16] and Yaman [17,18] utilized salicylic and ascorbic acid, UV stress, and combinations of TDZ and picloram to trigger callogenesis and metabolite enhancement.

Root cultures of *H. perforatum* have been extensively developed using IBA as the primary auxin. Brasili et al. [19] and Gaid et al. [20] used liquid MS media supplemented with IBA and glucose, further stimulated by chitosan or rotary shaking, while Valletta et al. [19] employed multi-hormonal treatments including 2,4-D, KIN, and NAA, plus chitosan dissolved in acetic acid. Red light, darkness, and UV-B exposure were also explored as elicitors [21,22]. Hairy root cultures were induced in *H. tomentosum* and *H. perforatum* using hormone-free or BA-enriched MS-B5 media [23,24].

Cell suspension cultures were established from callus using 2,4-D and BA, with methyl jasmonate or Ag nanoparticles further enhancing flavonoid or xanthone production [25,26]. Nano-perlite, MnO_2_ nanocomposites, and silver nanoparticles were tested in various shoot and callus cultures as abiotic elicitors, aiming to boost hypericin content [18,27].

*H. heterophyllum* was explored for both shoot and callus cultures using complex factorial designs of TDZ, IBA, and BAP, often in LS media, with abscisic and salicylic acids as additional elicitors [28]. Despite these advances, most protocols remain species-specific and highly dependent on genotype, medium composition, and treatment conditions, highlighting the need for continued optimization and comparative evaluation across taxa.

Based on the previously mentioned reports, in vitro shoot cultures appear to be a promising method for producing specific compounds, particularly hypericins, which primarily accumulate in the dark glands of the plant’s aerial parts. However, optimizing a reproducible protocol for each *Hypericum* species is essential, and further research is necessary [29].

Therefore, the present study aimed to develop an efficient in vitro propagation system for seven *Hypericum* species by evaluating the effects of two cytokinins [6-benzyladenine (BA) and meta-topoline (mT)] and two distinct culture periods (40 and 60 days) on biomass production. Additionally, the accumulation of bioactive compounds was analyzed using HPLC-DAD-MS-ESI to assess the impact of culture conditions on secondary metabolite profiles.

## 2. Results and Discussion

### 2.1. In Vitro Biomass Production

#### 2.1.1. In Vitro Culture Initiation and Aseptic Culture Establishment

Sterilized seeds placed on MS culture medium without plant growth regulators (PGRs) exhibited germination percentages ranging from 33.33% in *H. hirsutum* to 53.33% in *H. olympicum*. Intermediate values were recorded for *H. androsaemum* (36.67%), *H. calycinum* (40.00%), *H. kalmianum* and *H. perforatum* (both 46.67%), and *H. triquetrifolium* (36.67%). These results provided sufficient shoots to establish stable in vitro cultures for all species. Similar results on in vitro culture initiation from seeds have been reported for various *Hypericum* species, including *H. perforatum* [30], *H. hirsutum* and *H. maculatum* [31], *H. richeri ssp. transsilvanicum* and *H. umbellatum* [32], *H. triquetrifolium* [33], *H. aviculariifolium* and *H. pruinatum* [12], as well as *H. androsaemum*, *H. linariifolium, H. elodes, H. pulchrum*, *H. humifusum*, *H. undulatum*, *H. perfoliatum*, *H. canariense*, *H. tomentosum*, *H. perforatum,* and *H. maculatum* [2], *H. amblysepalum* [14], and *H. perforatum*, *H. leptophyllum*, and *H. heterophyllum* [34].

During the culture stabilization phase on MS medium without PGRs, the seven *Hypericum* species displayed species-specific morphogenetic behavior in terms of shoot number and length, but no rooting was observed in any of the species. On the PGR-free MS medium, well-branched plants were observed for *H. olympicum*, followed by *H. kalmianum*, *H. calycinum*, and *H. androsaemum*. In contrast, *H. hirsutum*, *H. perforatum*, and *H. triquetrifolium* produced more numerous, yet shorter and unbranched shoots.

Following subculturing onto MS medium supplemented with 0.1 mg/L BA, all species exhibited vigorous proliferation, forming compact and well-structured shoot clusters.

#### 2.1.2. Effect of Cytokinins and Culture Duration on In Vitro Biomass Production

To assess the effect of different cytokinins on biomass production in seven *Hypericum* species, they were cultivated on MS media supplemented with varying concentrations of BA and mT. Plantlets were subcultured and grown over two intervals (40 and 60 days) to evaluate both short- and medium-term responses.

When cultivated in vitro for 40 and 60 days, the seven *Hypericum* species showed variable growth and biomass accumulation responses on MS culture medium supplemented with different concentrations of BA and mT. The presence of BA in the culture medium significantly influenced biomass production, with MS + 0.2 mg/L BA yielding the highest biomass accumulation for most species (Table 1).

**Figure 1:**
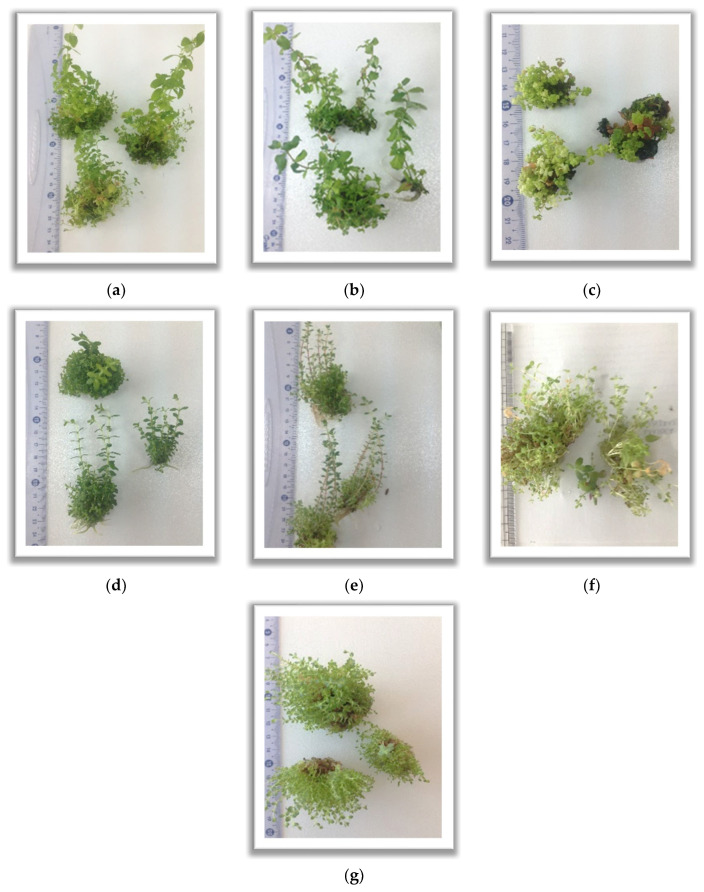
Morphology of cluster shoots from *Hypericum* species in vitro cultures in Murashige and Skoog medium supplemented with 0.2 mg/L 6-benzyladenine after a 60-day growth cycle: (**a**)—*H. androsaemum*; (**b**)—*H. calycinum*; (**c**)—*H. hirsutum*; (**d**)—*H. kalmianum*; (**e**)—*H. olimpicum*; (**f**)—*H. perforatum*; (**g**)—*H. triquetrifolium*.

Compared to MS without PGRs, the increase was significant and species-dependent. For example, in *H. perforatum*, biomass on MS + 0.2 mg/L BA reached 5.47 ± 0.97 g after 60 days—approximately 274 times higher than on MS without PGRs (0.02 ± 0.001 g). Similarly, *H. triquetrifolium* yielded 7.93 ± 0.34 g on MS + 0.2 mg/L BA versus 0.03 ± 0.01 g on MS without PGRs, representing a ~264-fold increase (Table 1). Figure 1 illustrates the general growth status of the seven *Hypericum* species under the most effective hormone treatment (MS + 0.2 mg/L BA) after 60 days of culture.

MS + 0.1 mg/L BA also significantly promoted in vitro shoot growth. Compared to MS without PGRs, *H. androsaemum* produced 115 times more biomass after 60 days (3.44 ± 0.35 g vs. 0.03 ± 0.01 g), *H. perforatum* nearly 100 times more (1.98 ± 0.40 g vs. 0.02 ± 0.001 g), and *H. triquetrifolium* 196 times more (5.89 ± 0.45 g vs. 0.03 ± 0.01 g).

As shown in Table 1, when comparing MS + 0.2 mg/L BA with MS + 0.1 mg/L BA, a significant increase was observed in *H. perforatum,* where biomass nearly tripled (5.47 ± 0.97 g vs. 1.98 ± 0.40 g). By contrast, in *H. olympicum*, the difference was minor (2.47 ± 0.22 g vs. 2.02 ± 0.27 g). In *H. triquetrifolium*, the recorded values for biomass production in MS + 0.2 mg/L BA, compared to MS + 0.1 mg/L BA, were higher, indicating a 35% increase (7.93 ± 0.34 g vs. 5.89 ± 0.45 g).

Media supplemented with mT did not stimulate biomass accumulation as effectively as BA. Even at higher concentrations (2 mg/L), mT had a limited impact, indicating that it is not effective for biomass production in the analyzed *Hypericum* species. On MS + 1 mg/L mT, biomass accumulation after 60 days was minimal across all species. For instance, *H. perforatum* produced only 0.02 ± 0.01 g, which is similar to MS without PGRs. In *H. triquetrifolium*, the biomass was 0.01 ± 0.01 g, showing no statistically significant differences from the PGR-free control. In *H. kalmianum*, 0.10 ± 0.01 g was recorded, which was significantly lower than the 4.09 ± 0.33 g obtained on MS + 0.2 mg/L BA (Table 1).

According to the data in Table 1, biomass increased slightly at MS + 2 mg/L mT compared to MS + 1 mg/L mT but remained low in comparison to the BA treatments. For instance, *H. perforatum* yielded 0.10 ± 0.01 g, which is 54 times lower than the 5.47 ± 0.97 g obtained with MS + 0.2 mg/L BA. In contrast, *H. triquetrifolium* produced only 0.07 ± 0.02 g, which is 113 times lower.

Plant height did not directly correlate with biomass accumulation. For instance, in *H. androsaemum*, shoot height on MS + 0.1 mg/L BA after 60 days was 8.41 ± 0.84 cm, which is 3.2 times higher than that on MS without PGRs (2.59 ± 0.48 cm). However, the biomass was not as high as that on MS + 0.2 mg/L BA, where the plants were shorter (2.68 ± 0.14 cm) but produced nearly three times more biomass (Table 2). These results suggest that balancing shoot elongation and proliferation is the key to maximizing biomass yield.

Extending the culture period from 40 to 60 days resulted in significantly increased biomass, particularly in BA-enriched media. In *H. perforatum*, biomass on MS + 0.2 mg/L BA increased by 1.42-fold, from 3.85 ± 0.64 g (40 days) to 5.47 ± 0.97 g (60 days). In *H. triquetrifolium*, it doubled (3.81 ± 0.38 g to 7.93 ± 0.34 g), while in *H. olympicum,* it increased nearly fourfold (0.62 ± 0.06 g to 2.47 ± 0.22 g). These data confirm that a longer culture period benefits biomass accumulation, although the extent of the effect is species-dependent. These findings are consistent with those of [35], who demonstrated that prolonging the culture period up to 80 days led to increased shoot proliferation and elongation in *H. perforatum* grown on semisolid medium. This reinforces the notion that extending the in vitro culture period can significantly enhance biomass yield.

As shown in Table 3, the 60-day culture period resulted in an overall decrease in shoot water content, possibly due to increased biomass and depletion of nutrients in the culture medium. This trend was most evident on MS without PGRs, where all species had significantly lower water content after 60 days compared to 40 days. For example, *H. hirsutum* showed a decrease from 81.80 ± 0.78% (40 days) to 71.04 ± 0.97% (60 days), and *H. androsaemum* from 86.18 ± 0.90% to 80.85 ± 0.73%.

Although in vitro plant cultures present a promising alternative for the efficient production of bioactive secondary metabolites due to their rapid initiation, independence from environmental factors, and ability to produce novel compounds, they are significantly affected by the type and concentration of plant growth regulators. For example, cytokinins, such as BA, play essential roles in regulating shoot proliferation and secondary metabolism in plants [1,36,37]. The effectiveness of BA (alone or in combination with other cytokinins or auxins) for shoot proliferation in various *Hypericum* species has been demonstrated in previous studies: *H. perforatum* [38,39,40], *H. maculatum* [41], *H. heterophyllum* [17], *H. scabroides* [42], *H. spectabile* [43], as well as *H. androsaemum*, *H. elodes*, *H. humifusum*, *H. linariifolium*, *H. perfoliatum*, *H. pulchrum*, *H. undulatum*, *H. canariense*, *H. tomentosum*, *H. maculatum*, and *H. perforatum* [2].

By contrast, mT, a naturally occurring cytokinin isolated from poplar leaves [44] has been scarcely used for *Hypericum* micropropagation. Meyer et al. [45] reported that treatment with 5 mM mT + 5 mM IAA induced 10 shoots per explant in *Hypericum* H2003-004-016 (a hybrid of *H. frondosum*, *H. galioides*, and *H. kalmianum*). Similar to our findings (especially in *H. olympicum* and *H. kalmianum*), the authors observed longer and thicker shoots with mT compared to BA treatments. Therefore, mT typically produces higher-quality plants, with better coloration and reduced incidence of morphological abnormalities during in vitro propagation [44]. Although mT tends to produce higher-quality shoots with fewer physiological disorders, its effect on biomass accumulation may be lower than that of BA. This is likely due to the rapid metabolic inactivation of mT through N9-glucosylation, leading to reduced receptor binding and shorter hormonal activity in planta [46].

**Table 1:** Effect of cytokinin and culture period on in vitro biomass accumulation in seven *Hypericum* species.

Treatment	*H. androsaemum*	*H. calycinum*	*H. hirsutum*	*H. kalmianum*	*H. olympicum*	*H. perforatum*	*H. triquetrifolium*
40 days							
MS	0.03 ± 0.01 a *	0.11 ± 0.02 a	0.03 ± 0.01 a	0.04 ± 0.01 a	0.08 ± 0.01 a	0.02 ± 0.001 a	0.01 ± 0.001 a
MS + 0.1 mg/L BA	0.95 ± 0.19 c	0.83 ± 0.21 b	0.56 ± 0.05 b	0.48 ± 0.14 a	1.00 ± 0.13 c	1.71 ± 0.26 b	3.41 ± 0.28 b
MS + 0.2 mg/L BA	0.32 ± 0.06 ab	0.35 ± 0.03 a	0.49 ± 0.06 b	0.45 ± 0.07 a	0.62 ± 0.06 bc	3.85 ± 0.64 c	3.81 ± 0.38 b
MS + 1 mg/L mT	0.02 ± 0.01 a	0.07 ± 0.01 a	0.03 ± 0.01 a	0.08 ± 0.01 a	0.06 ± 0.01 a	0.01 ± 0.001 a	0.01 ± 0.003 a
MS + 2 mg/L mT	0.14 ± 0.03 a	0.08 ± 0.01 a	0.08 ± 0.01 a	0.21 ± 0.02 a	0.19 ± 0.02 a	0.04 ± 0.01 a	0.03 ± 0.01 a
60 days							
MS	0.03 ± 0.01 a	0.13 ± 0.02 a	0.05 ± 0.01 a	0.16 ± 0.04 a	0.09 ± 0.01 a	0.02 ± 0.001 a	0.03 ± 0.01 a
MS + 0.1 mg/L BA	3.44 ± 0.35 e	1.90 ± 0.35 d	1.38 ± 0.19 c	2.16 ± 0.54 b	2.02 ± 0.27 d	1.98 ± 0.40 b	5.89 ± 0.45 c
MS + 0.2 mg/L BA	2.64 ± 0.24 d	1.20 ± 0.23 c	1.34 ± 0.16 c	4.09 ± 0.33 c	2.47 ± 0.22 e	5.47 ± 0.97 d	7.93 ± 0.34 d
MS + 1 mg/L mT	0.07 ± 0.02 a	0.13 ± 0.02 a	0.03 ± 0.01 a	0.10 ± 0.01 a	0.11 ± 0.02 a	0.02 ± 0.01 a	0.01 ± 0.01 a
MS + 2 mg/L mT	0.87 ± 0.15 bc	0.18 ± 0.03 a	0.13 ± 0.03 a	0.23 ± 0.03 a	0.44 ± 0.07 a	0.10 ± 0.01 a	0.07 ± 0.02 a

* Results are expressed in grams ± standard error. Values in one column marked with different lower cases denoted significant differences between samples at *p* < 0.05.

**Table 2:** Effect of cytokinin concentration and culture period on in vitro shoot clump height in seven *Hypericum* species.

Treatment	*H. androsaemum*	*H. calycinum*	*H. hirsutum*	*H. kalmianum*	*H. olympicum*	*H. perforatum*	*H. triquetrifolium*
40 days							
MS	2.21 ± 0.37 ab *	2.52 ± 0.20 ab	3.62 ± 0.35 e	2.84 ± 0.26 a	6.03 ± 0.58 b	3.24 ± 0.61 a	0.99 ± 0.25 ab
MS + 0.1 mg/L BA	5.81 ± 0.55 c	4.69 ± 0.43 d	0.89 ± 0.09 a	3.96 ± 0.38 b	4.09 ± 0.19 a	4.85 ± 0.29 b	2.41 ± 0.14 ef
MS + 0.2 mg/L BA	2.61 ± 0.19 ab	2.31 ± 0.24 a	1.43 ± 0.10 bc	2.60 ± 0.16 a	3.45 ± 0.27 a	3.04 ± 0.38 a	1.81 ± 0.16 cd
MS + 1 mg/L mT	1.62 ± 0.28 a	3.04 ± 0.33 bc	3.02 ± 0.16 d	4.58 ± 0.24 c	6.57 ± 0.32 b	2.61 ± 0.94 a	0.84 ± 0.34 ab
MS + 2 mg/L mT	3.15 ± 0.25 b	2.91 ± 0.22 abc	1.33 ± 0.13 ab	5.30 ± 0.26 de	12.28 ± 0.65 d	4.27 ± 0.64 b	1.31 ± 0.20 bc
60 days							
MS	2.59 ± 0.48 ab	3.24 ± 0.32 c	2.89 ± 0.18 d	4.91 ± 0.44 cd	9.64 ± 1.08 c	2.77 ± 0.66 a	2.79 ± 0.27 f
MS + 0.1 mg/L BA	8.41 ± 0.84 d	7.07 ± 0.70 e	1.87 ± 0.24 c	5.47 ± 0.40 e	8.55 ± 0.84 c	8.96 ± 1.19 e	4.56 ± 0.29 g
MS + 0.2 mg/L BA	2.68 ± 0.14 b	3.18 ± 0.22 bc	0.92 ± 0.11 a	2.60 ± 0.12 a	3.54 ± 0.17 a	3.04 ± 0.12 a	0.43 ± 0.02 a
MS + 1 mg/L mT	2.38 ± 0.24 ab	3.30 ± 0.15 c	2.69 ± 0.22 d	5.18 ± 0.27 de	5.98 ± 0.70 b	6.19 ± 1.12 c	1.25 ± 0.25 bc
MS + 2 mg/L mT	5.23 ± 0.49 c	3.51 ± 0.41 c	1.35 ± 0.19 ab	5.51 ± 0.32 e	16.30 ± 0.64 e	7.24 ± 0.87 d	1.97 ± 0.35 de

* Results are expressed in cm ± standard error. Values in one column marked with different lower cases denoted significant differences between samples at *p* < 0.05

**Table 3:** Effect of cytokinin and culture period on shoot water content in seven *Hypericum* species.

Treatment	*H. androsaemum*	*H. calycinum*	*H. hirsutum*	*H. kalmianum*	*H. olympicum*	*H. perforatum*	*H. triquetrifolium*
40 days							
MS	86.18 ± 0.90 c *	83.50 ± 1.17 b	81.80 ± 0.78 c	85.79 ± 0.62 de	84.67 ± 0.59 b	83.43 ± 1.46 c	88.00 ± 1.64 d
MS + 0.1 mg/L BA	90.28 ± 1.01 d	88.62 ± 0.97 c	88.80 ± 0.38 de	85.39 ± 1.46 de	86.15 ± 0.36 cd	90.47 ± 1.04 de	93.77 ± 0.15 ab
MS + 0.2 mg/L BA	90.20 ± 0.44 d	89.11 ± 0.28 cd	87.69 ± 1.43 d	89.89 ± 0.85 f	88.81 ± 0.41 e	92.49 ± 0.97 ef	93.57 ± 0.22 e
MS + 1 mg/L mT	87.65 ± 0.54 c	81.20 ± 1.85 b	80.76 ± 1.17 c	83.83 ± 0.48 cd	85.90 ± 0.81 bcd	80.49 ± 0.87 bc	81.55 ± 1.18 bc
MS + 2 mg/L mT	83.31 ± 1.06 b	76.48 ± 1.30 a	75.66 ± 1.02 b	80.77 ± 0.44 ab	83.32 ± 0.60 a	75.04 ± 0.88 a	79.77 ± 1.69 ab
60 days							
MS	80.85 ± 0.73 a	81.14 ± 0.65 b	71.04 ± 0.97 a	81.47 ± 0.77 ab	82.61 ± 0.63 a	79.12 ± 0.92 b	79.87 ± 1.69 ab
MS + 0.1 mg/L BA	91.55 ± 0.26 d	86.86 ± 1.28 c	85.69 ± 0.43 d	86.38 ± 1.41 e	87.13 ± 0.71 d	88.47 ± 0.74 d	93.31 ± 1.13 e
MS + 0.2 mg/L BA	95.21 ± 0.20 e	91.50 ± 0.64 d	91.42 ± 0.30 e	95.51 ± 0.11 g	91.67 ± 0.09 f	95.11 ± 0.20 f	96.12 ± 0.13 e
MS + 1 mg/L mT	87.20 ± 0.69 c	82.81 ± 0.86 b	79.75 ± 0.85 c	83.02 ± 0.55 bc	85.44 ± 0.29 bc	79.42 ± 2.02 b	83.51 ± 1.53 c
MS + 2 mg/L mT	86.19 ± 0.71 c	77.48 ± 1.14 a	75.77 ± 1.10 b	80.19 ± 0.52 a	85.58 ± 0.35 bc	78.22 ± 0.99 ab	78.13 ± 1.43 a

* Data are presented as mean water content (% FW) ± standard error. Values in one column marked with different lower cases denoted significant differences between samples at *p* < 0.05.

In this study, MS medium supplemented with 0.2 mg/L BA resulted in the highest biomass production, which justified the selection of biomass grown under this condition for further phytochemical analysis.

### 2.2. Analysis of Phenolic Compounds

#### 2.2.1. Identification and Classification of Phenolic Compounds

A total of 13 phenolic compounds were identified and quantified in the methanolic extracts of in vitro cultivated *Hypericum* species on MS culture medium supplemented with 0.2 mg/L BA for 40 and 60 days. The mass spectra, retention times, and UV-Vis absorption spectra were matched against reference standards, previously published data, and information from the Phenol-Explorer database to ensure a proper characterization of the phenolic compounds in the *Hypericum* extracts. These compounds belong to five major classes: hydroxycinnamic acids, anthocyanins, flavonols, phloroglucinols, and naphthodianthrones (Table 4). The anthocyanins cyanidin-glucoside and cyanidin-rhamnoside were not detected in *H. calycinum*, *H. kalmianum*, *H. olympicum*, and *H. triquetrifolium* at either culture period, indicating their absence in these species under the tested in vitro conditions.

**Figure 2:**
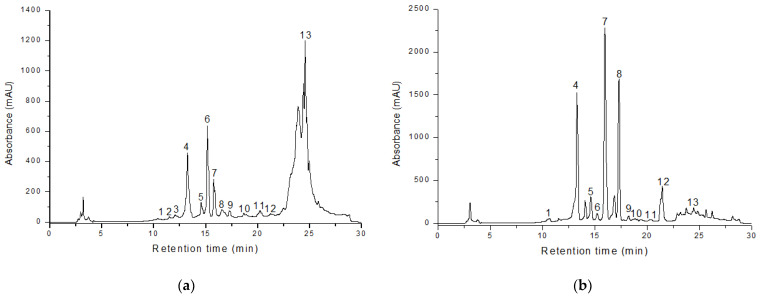
Representative HPLC-DAD profiles of methanolic extracts from (**a**) *H. olympicum* (60 days, highest hypericin content) and (**b**) *H. kalmianum* (60 days, highest total phenolic content). Peak numbers correspond to the identified compounds listed in Table 4.

Representative chromatograms obtained by HPLC-DAD-ESI-MS for two species—*H. olympicum* (60 days, highest hypericin content) and *H. kalmianum* (60 days, highest total phenolic content)—are shown in Figure 2. Chromatograms for the remaining species are available in Supplementary Figure S2.

#### 2.2.2. Quantitative Variation in Phenolic Content Among Species and Treatments

**Figure 3:**
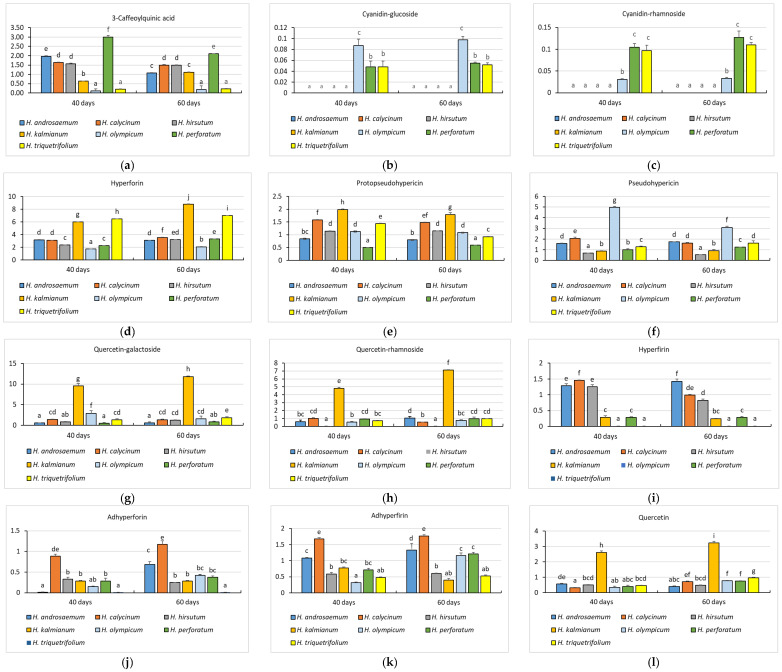

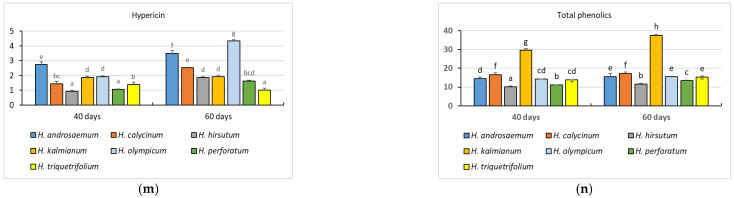
Individual phenolic compound content of seven *Hypericum* species biomass extracts cultivated in vitro for 40 and 60 days on MS + 0.2 mg/L BA: (**a**)—3-Caffeoylquinic acid; (**b**)—cyanidin-glucoside (**c**)—cyanidin-rhamnoside; (**d**)—hyperforin; (**e**)—protopseudohypericin; (**f**)—pseudohypericin; (**g**)—quercetin-galactoside; (**h**)—quercetin-rhamnoside; (**i**)—hyperfirin; (**j**)—adhyperforin; (**k**)—adhyperfirin; (**l**)—quercetin; (**m**)—hypericin; (**n**)—total phenolic compound. HPLC-DAD-MS-ESI was used for the analysis, and the values are expressed as milligrams per gram of dry weight ± standard error (mg/g DW ± SE). The bars marked with different lowercase letters denote significant differences between samples at *p* < 0.05.

*Hydroxycinnamic acids.* 3-Caffeoylquinic acid (Figure 3a) was the only hydroxycinnamic acid identified. It was most abundant in *H. perforatum* at 40 days (2.993 ± 0.093 mg/g), decreasing to 2.105 ± 0.010 mg/g at 60 days (−29.7%). This accumulation pattern is consistent with findings by [47], who also reported the highest content of this compound in *H. perforatum* grown under field conditions. A significant decrease in this compound was also observed in *H. androsaemum* (−45.3%), suggesting degradation over time. Conversely, *H. kalmianum* showed a strong increase from 1.736 ± 0.078 mg/g to 3.069 ± 0.099 mg/g (+76.7%), and *H. olympicum* increased by + 67.8%, indicating enhanced metabolic activity at 60 days.

*Anthocyanins.* Cyanidin-glucoside (Figure 3b) was detected only in *H. olympicum*, *H. perforatum*, and *H. triquetrifolium*. *H. olympicum* had the highest content (0.098 ± 0.006 mg/g at 60 days). Cyanidin-rhamnoside (Figure 3c) followed a similar distribution, peaking in *H. perforatum* (0.127 ± 0.015 mg/g at 60 days). Overall, anthocyanin content remained low in all species (<0.13 mg/g), but slightly increased over time in some taxa.

*Phloroglucinols.* Among the phloroglucinol derivatives, hyperforin (Figure 3d) showed the highest accumulation in *H. kalmianum* at 60 days (8.802 ± 0.047 mg/g), while *H. triquetrifolium* registered its peak at 40 days with 6.485 ± 0.006 mg/g. Protopseudohypericin (Figure 3e) reached a maximum concentration in *H. kalmianum* at 40 days (1.987 ± 0.018 mg/g) but showed a decline by 60 days. Pseudohypericin (Figure 3f) was most concentrated in *H. olympicum* at 40 days (4.966 ± 0.112 mg/g), followed by a notable reduction at 60 days (−37.7%). Hyperfirin (Figure 3i), on the other hand, exhibited the highest levels in *H. calycinum* (1.453 ± 0.012 mg/g at 40 days) and in *H. androsaemum* at 60 days (1.427 ± 0.071 mg/g), demonstrating a species-specific dynamic.

*Flavonols*. The flavonol subclass included quercetin-galactoside, quercetin-rhamnoside, and quercetin, all of which showed distinct species- and time-dependent accumulation patterns. Quercetin-galactoside (Figure 3g) was the most abundant compound in this group, with *H. kalmianum* reaching a peak concentration of 11.801 ± 0.151 mg/g at 60 days. This species also exhibited a robust increase in quercetin-galactoside content over time, while *H. perforatum* showed the highest relative increase (+63.3%) between the two cultivation periods. Quercetin-rhamnoside (Figure 3h) followed a similar trend, peaking again in *H. kalmianum* at 60 days (7.105 ± 0.053 mg/g), with limited variation across other species. Regarding quercetin itself (Figure 3l), its accumulation mirrored that of the other flavonols, with *H. kalmianum* recording the highest content (3.219 ± 0.115 mg/g at 60 days). These observations suggest that *H. kalmianum* possesses a highly active flavonol biosynthetic pathway, particularly under prolonged in vitro cultivation.

*Naphthodianthrones*. Within the class of naphthodianthrones, hypericin (Figure 3m) was the most abundant compound, especially in *H. olympicum*, where it reached a concentration of 4.334 ± 0.074 mg/g at 60 days. In contrast, adhyperforin (Figure 3j) and adhyperfirin (Figure 3k) were generally present in lower concentrations across all species, not exceeding 1.8 mg/g. Nonetheless, *H. calycinum* stood out as the most consistent producer of these two compounds, maintaining the highest levels relative to the other *Hypericum* taxa.

*Total phenolics*. Total phenolic content (Figure 3n) was highest in *H. kalmianum* (37.577 ± 0.433 mg/g at 60 days), followed by *H. calycinum* and *H. olympicum*. All species showed increased accumulation over time, with *H. kalmianum* exhibiting the largest gain (+26.6%). These results diverge from the decline observed in *H. perforatum* after 80 days of culture by [35], suggesting that 60 days represents an optimal harvest point before senescence affects biosynthesis.

The current results validate that phenolic compound accumulation is both species- and time-dependent. Compared to field conditions, in vitro cultivation not only maintained but also enhanced the biosynthetic capacity of lesser-known *Hypericum* species, including those with limited chemotypic profiles in the wild. Previous studies [48,49] support these findings, showing that in vitro systems, though costly, can compensate via higher phytochemical yields and allow for rapid propagation and biotechnological enhancement. Under our experimental conditions, *H. kalmianum*, *H. calycinum*, and *H. olympicum* emerged as leading candidates for future bioactive compound production.

According to Napoli et al. [47], hypericin was not detected in *H. androsaemum* or *H. calycinum*, and only trace amounts were found in *H. hirsutum*. Among the four species, only *H. perforatum* exhibited a significant hypericin content in wild conditions. In contrast, in our in vitro system, all four species accumulated detectable levels of hypericin, with *H. olympicum* and *H. hirsutum* surpassing *H. perforatum* in concentration. A similar trend was observed for pseudohypericin. This compound was absent in *H. androsaemum, H. calycinum*, and *H. hirsutum* in the field-grown samples reported by Napoli et al. [47]. However, under in vitro conditions, we found substantial accumulation of pseudohypericin in all four species, including those that showed no production in the wild. These results emphasize the influence of the in vitro environment, particularly controlled media composition and growth regulators, on activating the biosynthesis of pharmaceutically important naphthodianthrones, even in species not considered traditional producers.

In summary, the phytochemical analysis revealed distinct interspecific differences in total phenolic compound biosynthesis among the seven *Hypericum* species (Figure 3n). *H. kalmianum* clearly showed the highest potential, with the greatest increase in content (+26.6%) between 40 and 60 days (from 29.669 ± 0.703 mg/g to 37.577 ± 0.433 mg/g). In contrast, *H. hirsutum* had the lowest accumulation (10.140 ± 0.475 mg/g at 40 days and 11.608 ± 0.456 mg/g at 60 days). All species showed significant increases over time, suggesting that extending the in vitro culture period to 60 days can enhance phenolic compound accumulation. Our results contrast with those of [35], who reported a decline in total phenolic compounds in *H. perforatum* after 80 days of culture. This suggests that extending the culture beyond 60 days may lead to tissue senescence and decreased metabolic activity. As reported by Savio et al. [35], the decrease in phenolic levels at 80 days was associated with elevated PPO activity, indicating increased oxidative stress in aged cultures. This enzymatic shift may reflect a physiological transition from active growth to stress adaptation, leading to phenolic degradation. In our study, phenolic content increased steadily from 40 to 60 days in all species, indicating that 60 days may represent an optimal culture period for maximizing phenolic accumulation in *Hypericum* species under the tested conditions.

**Figure 4:**
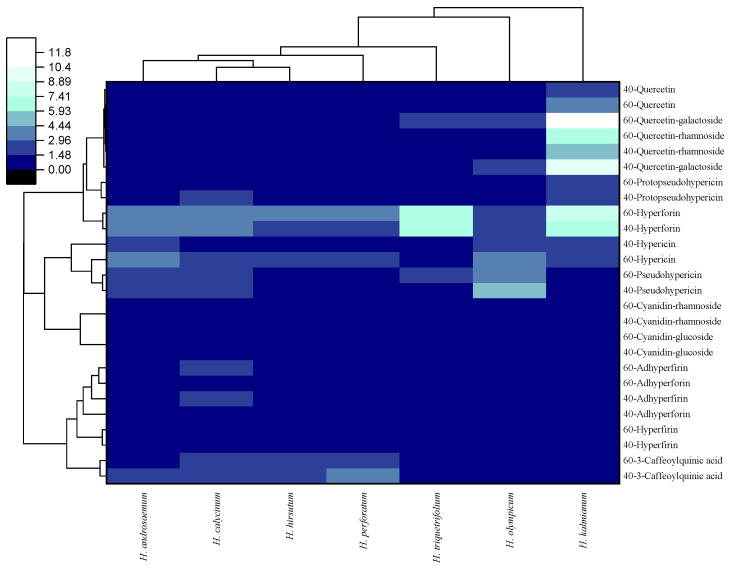
Heat map showing the content of each phenolic compound in the biomass extracts of seven *Hypericum* species at two different culture periods (40 and 60 days) on MS + 0.2 mg/L BA. The color intensity in each cell corresponds to the concentration of the respective phenolic compound, with a gradient ranging from black (lowest concentration) to red (intermediate concentration) to purple/blue (highest concentration).

The hierarchical cluster analysis illustrates the relationships among the *Hypericum* species and their phenolic compound profiles based on Euclidean distances. As shown in the heat map (Figure 4), distinct clustering patterns emerged, highlighting species with high or low accumulation of phenolic compounds during different in vitro culture periods (40 days vs. 60 days). *H. kalmianum* can be considered as an outlier, marked by a range of blue tones in the cells of the heat map. This indicates a distinct phenolic profile compared to that of other analyzed *Hypericum* species. The high content of phenolic compounds (37.57 ± 0.43 mg/g at 60 days) certainly contributes to this distinction. The following species—*H. androsaemum*, *H. calycinum*, *H. hirsutum*, and *H. perforatum*—indicated some similarities in their phenolic profiles. As shown in Figure 4, these species generally exhibit medium to low concentrations of the analyzed compounds, with a predominance of variation in the red hue of their profiles. These results suggest that the accumulation of phenolic compounds is influenced by both the species and the culture period, with *Hypericum* species displaying significant advantages when cultured for 60 days, such as *H. kalmianum* and *H. triquetrifolium*. This grouping mode may also be influenced by how phylogenetic relationships and ecological adaptations have shaped the diversification and chemical profiles of *Hypericum* species, as reported in previous studies [50,51].

Based on the relationships among phenolic compounds accumulated in vitro on MS medium supplemented with 0.2 mg/L BA, several *Hypericum* species are notable: flavonoids (quercetin, quercetin-rhamnoside, and quercetin-galactoside) were the most abundant biocompounds in *H. kalmianum*, underscoring its importance as a species with various therapeutic applications. Phloroglucinols (hyperforin, hyperfirin, adhyperforin, and adhyperfirin) were quantified in high concentrations in *H. kalmianum*, *H. triquetrifolium*, and *H. calycinum*, demonstrating the significant potential of these species for use in plant-based pharmaceutical products. Naphthodianthrones (hypericin, pseudohypericin, and protopseudohypericin) were primarily accumulated in *H. olympicum*, *H. calycinum*, and *H. hirsutum*, affirming the pharmaceutical value of these species. The results obtained in this study confirm the significant potential of in vitro systems for the biosynthesis of valuable compounds, while highlighting noteworthy variations among the seven *Hypericum* species cultivated under identical conditions.

Although previous studies have indicated that in vitro cultures of *Hypericum* are more costly than field or greenhouse cultivation, they offer substantial advantages such as the potential for genetic transformation and clonal propagation. Furthermore, their rapid biomass production, which correlates with yields of phytochemical compounds, can offset the high production costs, making this method competitive for producing high-quality products with multipurpose applications [48,49].

## 3. Materials and Methods

### 3.1. In Vitro Propagation

#### 3.1.1. Culture Medium and Culture Conditions

In this study, Murashige and Skoog (MS) medium [52] was used in all experiments. The carbon source was 30 g/L sugar (Margaritar, AGRANA Romania S.R.L., Roman, Romania). The pH of the medium was adjusted to 5.8 using 0.1 M NaOH and/or 0.1 M HCl before adding the gelling agent. Plant agar (5 g/L, *w*/*v*) was used to solidify the medium, followed by autoclaving at 121 °C for 20 min. Cultures were grown in 320 mL jars (6 cm diameter, 12 cm height) with transparent polypropylene lids, each containing 50 mL of medium. The in vitro cultures were incubated in a growth chamber under a 16-h photoperiod with a light intensity of 32.4 μmol m^−2^s^−1^ (cool white fluorescent light, Philips, Signify Romania S.R.L., Bucharest, Romania) and a temperature of 21 ± 3 °C. All chemicals were purchased from Duchefa Biochemie B.V., Haarlem, Netherlands.

#### 3.1.2. Initiation and Stabilization of In Vitro Cultures

For in vitro culture initiation, seeds from seven *Hypericum* species were used: *Hypericum androsaemum* L., *Hypericum calycinum* L., *Hypericum hirsutum* L., *Hypericum kalmianum* L., *Hypericum olympicum* L., *Hypericum perforatum* L., and *Hypericum triquetrifolium* Turra. The seeds were washed under running tap water for 15 min to remove dust and impurities, then decontaminated with a 20% ACE bleach solution (Procter & Gamble, Romania S.R.L, Urlati, Romania; <5% active ingredient) for 15 min. Subsequently, the ACE solution was diluted to 10% with sterile water, and the disinfection process continued for another 5 min. Without rinsing to remove chlorine traces, the seeds were inoculated on MS medium without plant growth regulators (PGRs), with ten seeds per jar in three jars per species. To stabilize the in vitro cultures, shoots obtained after 20 days were cut into 1.0–1.5 cm fragments and cultivated in the same medium, with five shoot fragments per jar, for 30 days. Next, the shoots were subcultured onto MS medium supplemented with 0.1 mg/L BA, with three shoot fragments per jar. After two 30-day subcultures, a sufficient number of shoots were obtained for further experiments.

#### 3.1.3. Biomass Production

For the biomass production study, shoots obtained from MS medium containing 0.1 mg/L BA were cut to a length of 1.5–2.0 cm and placed in 370 mL glass jars filled with 50 mL of MS basal medium, with or without cytokinins (three explants per jar in five jars per treatment per species). The effects of BA and mT on biomass production were evaluated for each *Hypericum* species at four different concentrations: 0.1 mg/L and 0.2 mg/L BA, and 1.0 mg/L and 2.0 mg/L mT. The results were compared to a PGR-free medium.

The growth response to cytokinins was assessed at 40 and 60 days using four parameters: the length of intact clumps (cm); the fresh weight (FW) of shoots per explant, measured immediately after removal from the in vitro medium; the dry weight (DW) of shoots, determined after drying the material for three days at 45 °C; and the water content (WC), calculated according to the formula: WC (%) = ((Fresh Weight − Dry Weight)/Fresh Weight) × 100.

### 3.2. HPLC-DAD-MS-ESI Analysis

The phenolic compounds were analyzed in plants grown in MS culture medium supplemented with 0.2 mg/L BA, with the highest biomass obtained for most *Hypericum* species after 40 days and 60 days of cultivation, respectively.

#### 3.2.1. Sample Preparation and Chromatographic Conditions

Phenolic compounds were analyzed from biomass of the seven *Hypericum* species grown on MS medium supplemented with 0.2 mg/L BA, which was identified as the most effective treatment for biomass accumulation. Samples were collected after 40 and 60 days of in vitro cultivation.

For extraction, 0.2 g of freeze-dried biomass was mixed with 2 mL of methanol acidified with 1% HCl (*v*/*v*, 37% stock solution), vortexed for 1 min, followed by 30 min of ultrasonic-assisted extraction at room temperature. The extract was centrifuged at 10,000 rpm for 10 min, and the supernatant was filtered through a 0.45 µm nylon membrane prior to HPLC injection.

Chromatographic analysis was carried out using an HP-1200 HPLC system (Agilent Technologies, Santa Clapa, CA, USA) equipped with a quaternary pump, autosampler, diode array detector (DAD), and a single quadrupole API-electrospray ionization mass spectrometer (MS-6110) (HPLC-DAD-MS-ESI). Phenolic separation was performed on a Kinetex XB-C18 column (5 µm, 4.6 × 150 mm; Phenomenex Inc., Torrance, CA, USA) maintained at 25 ± 0.5 °C.

The mobile phase consisted of solvent A (0.1% formic acid in water) and solvent B (0.1% formic acid in acetonitrile). A multistep linear gradient was applied as follows: 5% B for 2 min, increasing to 90% B over 20 min, held at 90% B for 4 min, and re-equilibrated to 5% B in 6 min. The total run time was 30 min at a flow rate of 0.5 mL/min. Chromatograms were recorded at multiple wavelengths (λ = 280, 340, and 520 nm).

#### 3.2.2. Chemical Reagents and Materials

HPLC-grade acetonitrile was purchased from Merck KGaA (Darmstadt, Germany), and deionized water was obtained from a Direct-Q UV system (Millipore Corporation, Billerica, MA, USA). Phenolic standards used for calibration included chlorogenic acid, rutin, and cyanidin chloride (Sigma-Aldrich, Saint Louis, MI, USA; ≥99% purity).

#### 3.2.3. Quantitative Determination

Compound quantification was based on the external standard method. Hydroxycinnamic acids were quantified as chlorogenic acid equivalents (R^2^ = 0.9937) in the linearity range 10–50 μg/mL; flavonols, phloroglucinols, and naphthodianthrones as rutin equivalents (R^2^ = 0.9981) in the linearity range 10–100 μg/mL; and anthocyanins as cyanidin equivalents (R^2^ = 0.9951). Results are expressed as milligrams per gram of dry weight (mg/g DW). (Calibration curve—Supplementary Figure S1).

### 3.3. Experimental Design

The in vitro experiments followed a factorial design with three factors: seven *Hypericum* species (*H. androsaemum*, *H. calycinum*, *H. hirsutum*, *H. kalmianum*, *H. olympicum*, *H. perforatum*, and *H. triquetrifolium*), five culture media [MS without PGRs, MS + 0.1 mg/L BA, MS + 0.2 mg/L BA, MS + 1 mg/L mT, MS + 2 mg/L mT], and two culture periods (40 and 60 days).

### 3.4. Data Analysis

Phenolic compound content was analyzed using HPLC-DAD-MS-ESI, and values are expressed as milligrams per gram of dry weight (mg/g DW).

Two-way ANOVA was performed to test for differences between experimental variants. When the null hypothesis was rejected, ANOVA was followed by Tukey’s HSD test (*p* < 0.05) to identify significant differences between means. Values are presented as mean ± S.E.

A heat map was used to visualize the variation in the content of 13 phenolic compounds across the seven *Hypericum* species at two growth stages (40 and 60 days) under MS + 0.2 mg/L BA culture conditions. The phenolic compounds were clustered using Euclidean distance.

OriginPro 2021 and Excel 2016 were used for statistical analysis and data visualization, respectively.

## 4. Conclusions

This research demonstrates the effectiveness of in vitro culture systems for enhancing biomass production and the biosynthesis of phenolic compounds in *Hypericum* species. The results also reveal the significant influence of the in vitro culture period on biomass and metabolite accumulation. Among the tested conditions, MS medium supplemented with 0.2 mg/L BA and a culture period of 60 days proved most effective for shoot proliferation and the accumulation of bioactive compounds. Notably, *H. kalmianum* showed particular promise as a source of total phenolics, while *H. olympicum* excelled in producing hypericin derivatives. *H. androsaemum* and *H. calycinum*, which are less investigated species than *H. perforatum*, exhibited distinctive phytochemical profiles under these in vitro conditions, suggesting their potential value for targeted compound extraction 6.

## Figures and Tables

**Table 4:** Identification of phenolic compounds detected in methanolic extracts of *Hypericum* species cultivated in vitro on MS medium with 0.2 mg/L BA. Compounds were separated by HPLC-DAD and identified by retention time (Rt), UV-Vis absorbance maxima (λmax), and mass spectrometry (ESI-MS) data in positive ionization mode ([M + H]^+^).

Peak No.	R_t_ (min)	UV λ_max_ (nm)	[M + H]^+^ (*m*/*z*)	Phenolic Compound	Subclass
1	10.34	332	355	3-Caffeoylquinic acid (Neochlorogenic acid)	Hydroxycinnamic acid
2	11.01	528	449.287	Cyanidin-glucoside	Anthocyanin
3	12.18	530	433.287	Cyanidin-rhamnoside	Anthocyanin
4	13.21	320	537	Hyperforin	Phloroglucinol
5	14.54	290	523	Protopseudohypericin	Naphthodianthrone
6	15.13	290	523	Pseudohypericin	Naphthodianthrone
7	15.83	360	465.303	Quercetin-galactoside	Flavonol
8	17.31	360	449.303	Quercetin-rhamnoside	Flavonol
9	17.71	322	467	Hyperfirin	Phloroglucinol
10	18.73	320	551	Adhyperforin	Phloroglucinol
11	20.06	322	481	Adhyperfirin	Phloroglucinol
12	21.05	360	303	Quercetin	Flavonol
13	24.42	330	505	Hypericin	Naphthodianthrone

## Data Availability

Data will be made available on request.

## Supplementary Materials

The following supporting information can be downloaded at: https://www.mdpi.com/article/10.3390/plants14152437/s1, Figure S1: HPLC chromatograms of *Hypericum* species after 40 and 60 days of in vitro culture: (a,b) *H. androsaemum* at 40 and 60 days, respectively; (c,d) *H. calycinum* at 40 and 60 days; (e,f) *H. hirsutum* at 40 days; (g,h) *H. perforatum* at 40 and 60 days; (i,j) *H. triquetrifolium* at 40 and 60 days; (k) *H. olympicum* at 40 days; (l) *H. kalmianum* at 40 days. Figure S2: HPLC chromatograms of *Hypericum* species after 40 and 60 days of in vitro culture: (a,b) *H. androsaemum* at 40 and 60 days, respectively; (c,d) *H. calycinum* at 40 and 60 days; (e,f) *H. hirsutum* at 40 days; (g,h) *H. perforatum* at 40 and 60 days; (i,j) *H. triquetrifolium* at 40 and 60 days; (k) *H. olympicum* at 40 days; (l) *H. kalmianum* at 40 days.
