# Supplementary material for: Effects of Culture Period and Plant Growth Regulators on In Vitro Biomass Production and Phenolic Compounds in Seven Species of Hypericum
Source: Plants (Basel). 2025 Aug 6;14(15):2437. doi: 10.3390/plants14152437 (PMC12349193; doi:10.3390/plants14152437)
Supplement: Supplementary file 1 [file plants-14-02437-s001.zip › Supplementary Figure S1_Calibration curve.pdf]

Supplementary Figure S1 - Calibration curves : (a)-The hydroxycinnamic acid content was calculated using a five-point calibration curve of chlorogenic acid (R^2=0.9937) in the linearity range10-50 µg/ml; (b) - The flavonol, phloroglucinol and naphthodianthrone content was calculated using a five-point calibration curve of rutin (R^2=0.9981) in the linearity range10-100 µg/ml; (c) - The anthocyanins content was calculated using a five-point calibration curve of cyaniding (R^2=0.9951) in the linearity range 10-100 µg/ml.

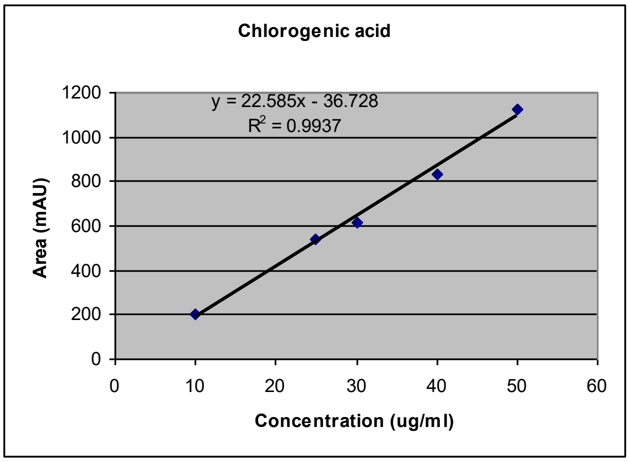

(a)

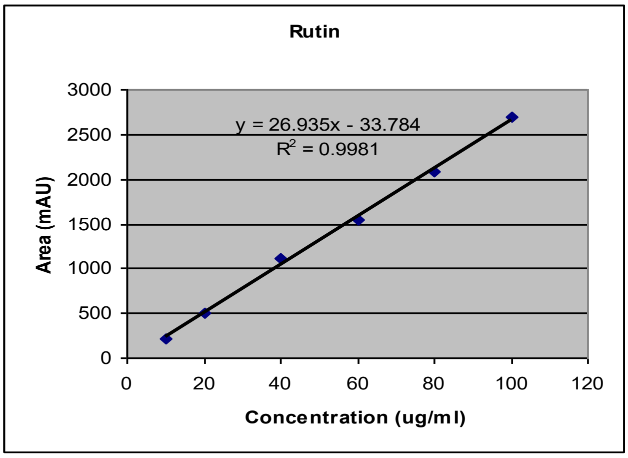

(b)

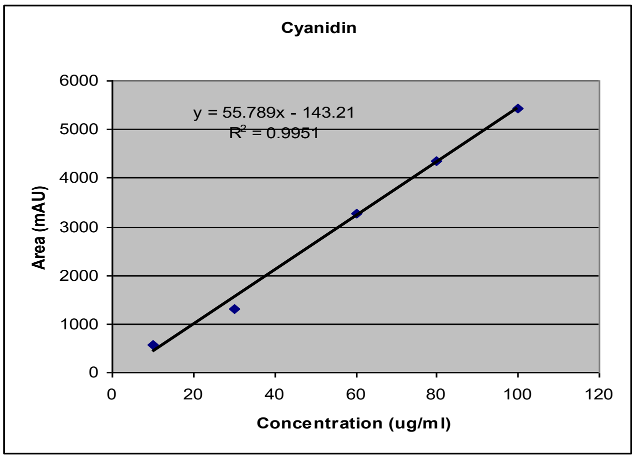

(c)
