# Supplementary material for: Effects of Culture Period and Plant Growth Regulators on In Vitro Biomass Production and Phenolic Compounds in Seven Species of Hypericum
Source: Plants (Basel). 2025 Aug 6;14(15):2437. doi: 10.3390/plants14152437 (PMC12349193; doi:10.3390/plants14152437)
Supplement: Supplementary file 1 [file plants-14-02437-s001.zip › Supplimentary Figure S2_HPLC chromatograms material.pdf]

Supplementary Figure S2. HPLC chromatograms of *Hypericum* species after 40 and 60 days of in vitro culture: (a, b) *H. androsaemum* at 40 and 60 days, respectively; (c, d) *H. calycinum* at 40 and 60 days; (e, f) *H. hirsutum* at 40 days; (g, h) *H. perforatum* at 40 and 60 days; (i, j) *H. triquetrifolium* at 40 and 60 days; (k) *H. olympicum* at 40 days; (l) *H. kalmianum* at 40 days.

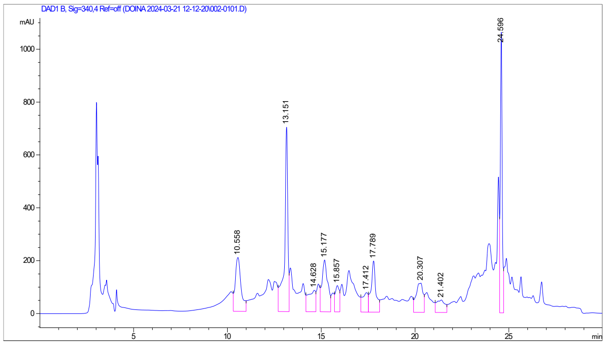

(a)

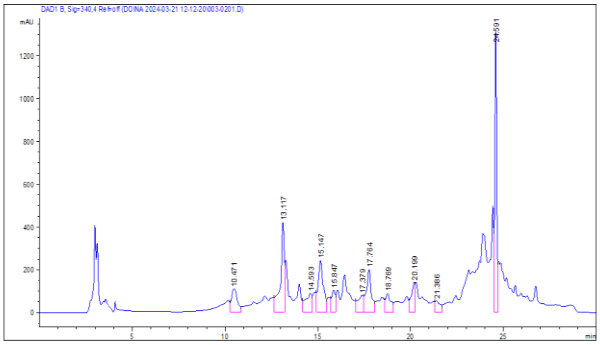

(b)

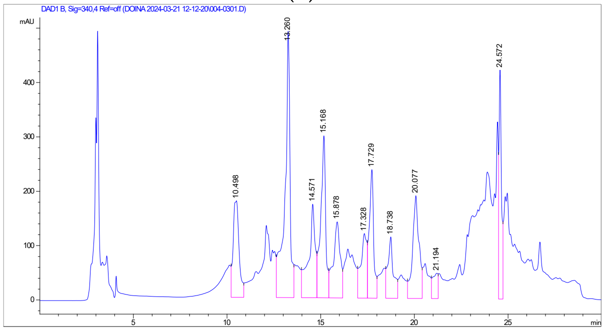

(c)

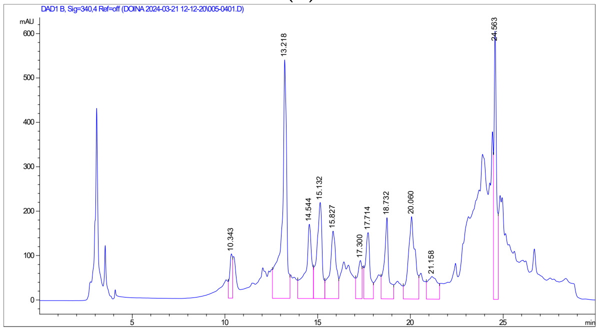

(d)

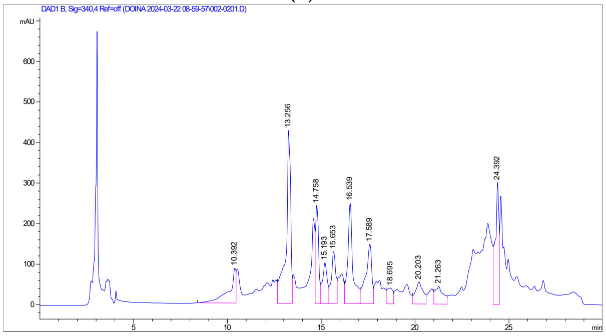

(e)

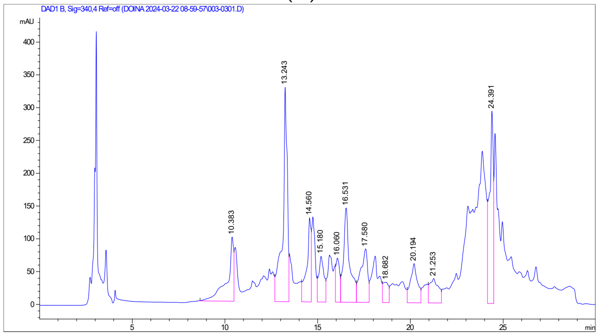

(f)

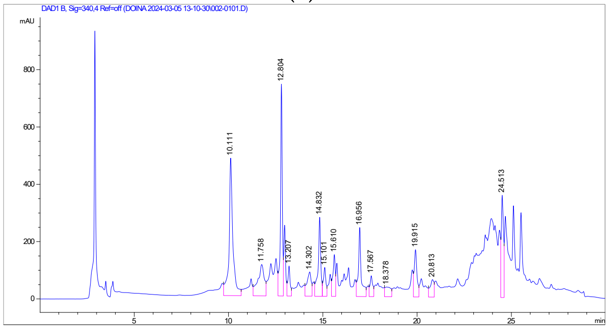

(g)

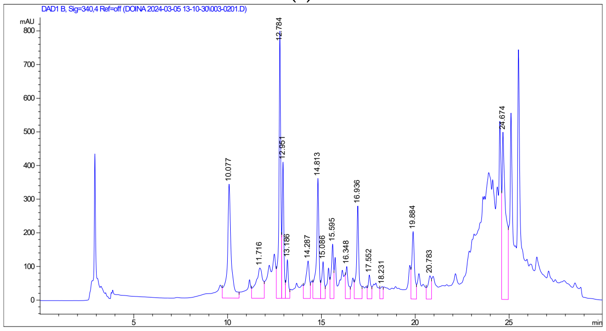

(h)

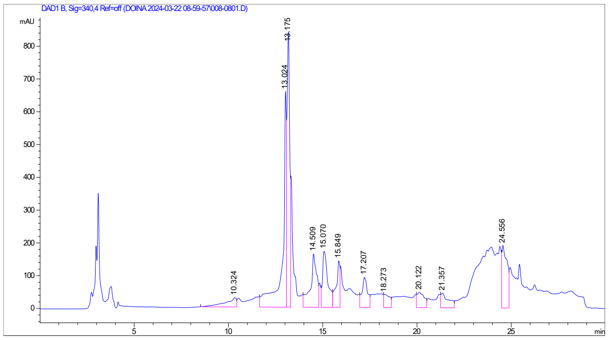
DAD1 B, Sig=340,4 Ref=off (DOINA 2024-03-22 08-59-57\008-0801.D)
mAU
10.324
13.024
13.175
14.509
15.070
15.849
17.207
18.273
20.122
21.357
24.556
min

(i)

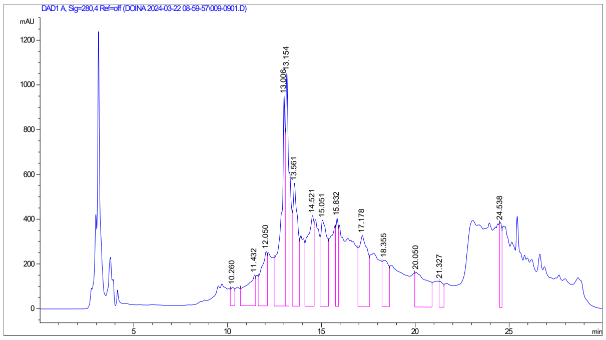
DAD1 A, Sig=280,4 Ref=off (DOINA 2024-03-22 08-59-57\009-0901.D)
mAU
10.260
11.432
12.050
13.006
13.154
13.561
14.521
15.051
15.832
17.178
18.355
20.050
21.327
24.538
min

(j)

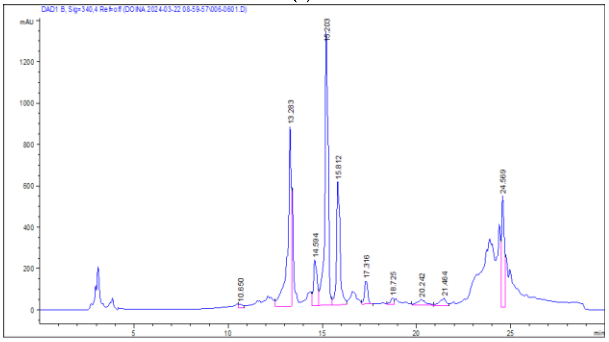
DAD1 B, Sig=340,4 Ref=off (DOINA 2024-03-22 08-59-57\006-0601.D)
mAU
10.650
13.283
14.594
15.203
15.812
17.316
18.725
20.242
21.464
24.569
min

(k)

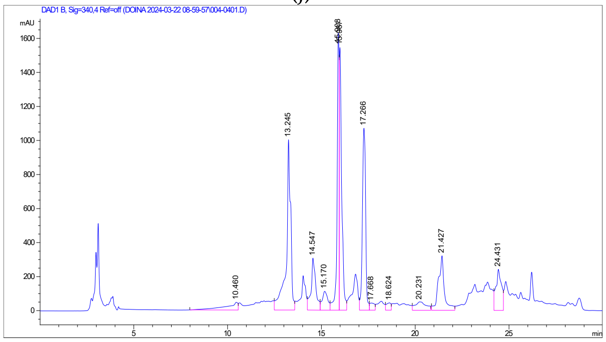
DAD1 B, Sig=340,4 Ref=off (DOINA 2024-03-22 08-59-57\004-0401.D)
mAU
10.460
13.245
14.547
15.170
[illegible]
[illegible]
17.266
17.668
18.624
20.231
21.427
24.431
min

(l)
